# Joint Extraction of Cyber Threat Intelligence Entity Relationships Based on a Parallel Ensemble Prediction Model

**DOI:** 10.3390/s25165193

**Published:** 2025-08-21

**Authors:** Huan Wang, Shenao Zhang, Zhe Wang, Jing Sun, Qingzheng Liu

**Affiliations:** 1 School of Computer Science and Technology, Guangxi University of Science and Technology, Liuzhou 545006, China; wanghuan@gxust.edu.cn (H.W.); 20230701020@stdmail.gxust.edu.cn (S.Z.); sunjing_gxust@gxust.edu.cn (J.S.); qingzhengliu@gxust.edu.cn (Q.L.); 2 Guangxi Colleges and Universities Key Laboratory of Intelligent Computing and Distributed Information Processing, Liuzhou 545006, China; 3 Guangxi Education System Network Security Monitoring Center, Liuzhou 545006, China

**Keywords:** cyber threat intelligence, parallel ensemble prediction, BiGRU, non-autoregressive decoder, entity–relationship extraction

## Abstract

The construction of knowledge graphs in cyber threat intelligence (CTI) critically relies on automated entity–relation extraction. However, sequence tagging-based methods for joint entity–relation extraction are affected by the order-dependency problem. As a result, overlapping relations are handled ineffectively. To address this limitation, a parallel, ensemble-prediction–based model is proposed for joint entity–relation extraction in CTI. The joint extraction task is reformulated as an ensemble prediction problem. A joint network that combines Bidirectional Encoder Representations from Transformers (BERT) with a Bidirectional Gated Recurrent Unit (BiGRU) is constructed to capture deep contextual features in sentences. An ensemble prediction module and a triad representation of entity–relation facts are designed for joint extraction. A non-autoregressive decoder is employed to generate relation triad sets in parallel, thereby avoiding unnecessary sequential constraints during decoding. In the threat intelligence domain, labeled data are scarce and manual annotation is costly. To mitigate these constraints, the SecCti dataset is constructed by leveraging ChatGPT’s small-sample learning capability for labeling and augmentation. This approach reduces annotation costs effectively. Experimental results show a 4.6% absolute F1 improvement over the baseline on joint entity–relation extraction for threat intelligence concerning Advanced Persistent Threats (APTs) and cybercrime activities.

## 1. Introduction

With the rapid development of internet technology, cybersecurity threats have become increasingly complex and diverse. Advanced Persistent Threats (APTs), ransomware, distributed denial-of-service (DDoS) attacks, and other emerging attack types pose serious risks to the critical infrastructures of enterprises and nations [1,2]. In this context, cyber threat intelligence (CTI) has been recognized as a key component of cyber defense and capability enhancement [3]. CTI is intended to provide timely and actionable strategies by collecting, analyzing, and sharing dynamic threat information. Nevertheless, the accurate mining and analysis of high-value intelligence from large volumes of CTI reports remain significant challenges for cybersecurity professionals [4].

The core of CTI knowledge mining lies in the accurate extraction and analysis of extensive information. This information includes attacker behaviors, vulnerabilities and exploits, defender actions, attack tools, and the organizational relationships behind adversaries. The effective management of such intelligence is essential for informed defense decision-making [5]. Knowledge graphs describe concepts, entities, and inter-entity relations in a structured form that aligns more closely with human cognition [6]. By leveraging knowledge graph-based information extraction techniques, large volumes of threat intelligence can be organized and interpreted more effectively. The primary tasks of information extraction are Named Entity Recognition (NER) and relation extraction (RE), whose objective is to identify entities and the relations expressed among them in text.

In the early stage of development, entity and relation extraction were primarily based on expert-crafted pattern-matching rules [7] and statistical machine learning methods [8]. However, these approaches depended on hand-engineered rules and features, which limited domain adaptability. With advances in artificial intelligence, rapid developments in Natural Language Processing (NLP) and deep learning have substantially advanced entity and relation extraction. Pipeline architectures were adopted for Named Entity Recognition (NER). In particular, deep models combining Recurrent Neural Networks (RNNs) with Conditional Random Fields (CRFs) were proposed [9]. These models captured contextual dependencies more effectively and improved NER accuracy.

After NER, attention-based Graph Neural Network (GNN) models were introduced for relation extraction [10]. The full dependency tree was used as input. Soft pruning was applied to automatically learn and retain informative substructures relevant to the relation extraction task. However, pipeline extraction suffers from error propagation: mistakes in upstream NER directly degrade downstream relation extraction accuracy.

To mitigate error propagation, information extraction has recently been modeled as a sequence-to-sequence (Seq2Seq) joint entity–relation extraction problem [11,12]. End-to-end attention mechanisms were introduced to capture word-level dependencies. Outputs were generated as sequences of tokens. However, performance degraded on complex sentences and long documents, and triples with overlapping entities were handled poorly.

To address the shortcomings of prior methods, a joint entity–relation extraction approach for cyber threat intelligence is proposed. The task is reformulated as an ensemble-prediction problem within a parallel framework. A joint network that combines BERT (Bidirectional Encoder Representations from Transformers) [13] and BiGRU (Bidirectional Gated Recurrent Unit) [14] is constructed to capture deep contextual features in sentences. An ensemble prediction module and an entity–relation triple representation are designed for joint extraction. A non-autoregressive decoder is employed to generate sets of relation triples in parallel, thereby avoiding unnecessary ordering constraints during sequence generation.

The main contributions of this work are summarized below:

1. A joint entity–relation extraction model for CTI is proposed. CTI triples are generated in parallel to mitigate the sequential-dependency issue inherent to sequence-labeling methods.
2. A cost-effective CTI dataset, SecCti, is constructed. ChatGPT’s small-sample capability is leveraged via Q&A prompts for data labeling and augmentation, and all annotations are verified by security professionals, substantially reducing manual labeling costs.
3. Improved robustness to overlapping-entity triples is demonstrated. An absolute F1 score of 89.1% is achieved in the corresponding evaluations.

## 2. Related Work

Information extraction from CTI reports primarily involves Named Entity Recognition (NER) and relation extraction (RE). Deep learning–based approaches are typically categorized into pipeline extraction and joint extraction.

In pipeline extraction, NER is performed first, followed by RE based on the recognized entities. The performance of RE is therefore highly dependent on NER quality. Gao et al. [15] proposed an NER model that combines a data-driven deep learning approach with a knowledge-driven dictionary method to construct dictionary features, improving the recognition of rare entities and complex words. Wang et al. [16] developed a neural unit, GARU, to integrate features from Graph Neural Networks (GNNs) and Recurrent Neural Networks (RNNs), alleviating fuzzy interactions between heterogeneous networks. To address data sparsity in CTI, Liu et al. [17] proposed a semantic enhancement method that encodes and aggregates compositional, morphological, and lexical features of input tokens to retrieve semantically similar words from a cybersecurity corpus. Sarhan et al. [18] devised a CTI knowledge-graph framework in which NER models are first trained to recognize entities, and relation triples generated by Open Information Extraction (OIE) are subsequently labeled. Although these methods perform well on standalone NER, RE performance remains constrained by NER quality, leading to error propagation.

Joint extraction aims to identify both entities and relations directly from CTI text, improving accuracy and efficiency in cyber threat monitoring and response. Because relation triples within a sentence may share overlapping entities, several joint models have been proposed. Yuan et al. [19] introduced a Relation-Specific Attention Network (RSAN) to jointly extract relations in sentences. RSAN combines relation-based attention with a gating mechanism over a Bidirectional Long Short-Term Memory (BiLSTM) network to produce relation-guided sentence representations, reducing redundant operations observed in pipeline methods. Building on relation-based attention, Guo et al. [11] formulated CTI entity–relation joint extraction as a multi-sequence labeling problem. Text features were extracted with a Bidirectional Gated Recurrent Unit (BiGRU), and decoding was performed using BiGRU and a Conditional Random Field (CRF). Zuo et al. [20] developed an end-to-end sequential labeling model based on BERT-att-BiLSTM-CRF for joint extraction, and knowledge triples were finally obtained using entity–relation matching rules. Ahmed et al. [12] improved upon Zuo et al. [20] by employing an attention-based RoBERTa-BiGRU-CRF model for sequential labeling, mitigating limitations of classical pipeline techniques. Despite these advances, traditional sequence-labeling approaches still struggle with overlapping entities due to order constraints and thus suffer from order dependency.

Recently, the non-autoregressive decoder (NAD) has been widely explored for natural language tasks [21]. Sui et al. [22] formulated joint entity–relation extraction as a set-prediction problem, relieving the model of the sequential burden of predicting multiple triples. Jun Yu et al. [23] applied an NAD to the aspect-level sentiment triad extraction (ASTE) task. The encoding layer used MPNet (proposed by Microsoft), and dependencies among predicted tokens were modeled via Permutation Language Modeling (PLM). Combined with the non-autoregressive decoder, this design achieved state-of-the-art results on ASTE. Yanfei Peng et al. [24] proposed an end-to-end model with a dual ensemble-prediction network to decode triples (termed “ternary groups” in their work). Entity pairs and relations were decoded sequentially, and interactions were strengthened through parameter sharing across the two ensemble-prediction networks. Additionally, entity filters were designed to tighten subject–object connections and to discard triples with low subject/object confidence.

## 3. Materials and Methods

In this study, unstructured cybersecurity threat intelligence in PDF format was collected from relevant sources. The data were cleaned and preprocessed to obtain sentence-level text. Relation triples present in the sentences were then manually annotated to serve as inputs to the model. The joint entity–relation extraction model consists of two core modules. First, sentences are encoded with a joint BERT–BiGRU encoder to capture sequence dependencies and to provide rich contextual information for the non-autoregressive decoder (NAD). Second, the NAD is used to generate predicted triples in parallel. The model is optimized with a bipartite matching loss, ensuring the best alignment between predicted triples and gold triples.

**Figure 1:**
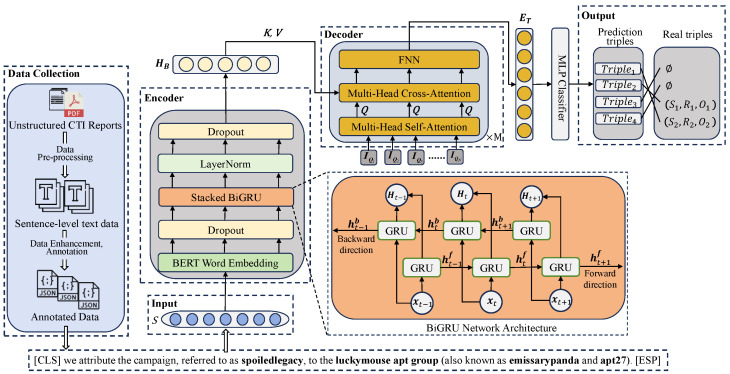
The methodological architecture of this paper.

The method architecture of this paper is shown in Figure 1, where the sentence $S=\{w_{1},w_{2},...,w_{t},...,w_{l}\}$ is the input to the model with length *l* (containing [CLS] and [SEP]), and the set of relation triples in the sentence is $T={\left\{\left(h_{i},r_{i},t_{i}\right)|h_{i},t_{i}\in E,r_{i}\in R\right\}}_{i=1}^{m}$, where *m* is the number of triples, and *E* and *R* are the set of entities and the set of relations, respectively; the specific examples are shown in Table 1. There exist two kinds of cases: one is the ordinary example, where there is no overlapping entity in the triple, and the other is the overlapped example, where the the sentence is more complex and there are multiple overlapping entities. A Bidirectional Gated Recurrent Unit (BiGRU) network input vector $x_{t}$ denotes the word embedding vector of $w_{t}$ after passing through the BERT layer; vector $H_{t}$ denotes the word feature representation of $x_{t}$ after passing through a layer of BiGRU encoding; vector $H_{B}$ denotes the output of the encoder; and ${\left\{I_{Q_{i}}\in R^{d}\right\}}_{i=1}^{N}$ denotes the learnable embeddings, which are used as initialization inputs to the decoder, where *N* is the number of learnable embeddings and *d* is the initial dimension of the BERT word embeddings. $E_{T}$ denotes the output of the decoder. $Triple_{1}$ denotes the final prediction of the obtained relational triple; the final goal of the entity–relationship joint extraction model is to extract the set of relational triples T present in the sentence *S*.

The overall architecture is shown in Figure 1. A sentence is denoted by $S=\{w_{1},w_{2},...,w_{t},...,w_{l}\}$ with length *l* (including [CLS] and [SEP]). The set of relation triples is defined as $T={\left\{\left(h_{i},r_{i},t_{i}\right)|h_{i},t_{i}\in E,r_{i}\in R\right\}}_{i=1}^{m}$, where *m* is the number of triples, and *E* and *R* denote the entity and relation sets, respectively. Examples are provided in Table 1. Two cases are considered. In the ordinary case, no entity overlap occurs within a triple. In the overlapped case, the sentence is more complex and contains multiple overlapping entities. For the encoder, $x_{t}$ is the BERT embedding of $w_{t}$. The vector $H_{t}$ is the contextual representation after a BiGRU layer, and $H_{B}$ is the encoder output. Learnable embeddings ${\left\{I_{Q_{i}}\in R^{d}\right\}}_{i=1}^{N}$ are used to initialize the decoder, where *N* is the number of learnable embeddings and *d* is the BERT embedding dimension. The decoder output is $E_{T}$. The final prediction of relation triples is denoted by $Triple_{1}$. The objective of the joint entity–relation extraction model is to recover the set T of relation triples present in *S*.

### 3.1. Entity Relationship Category Design

A relatively mature system of core concepts for cyber threat intelligence (CTI) and security elements has been established [25,26,27,28]. Based on this body of knowledge and the CTI corpus collected in this study, eleven entity categories were defined: operating system (OS), malware (MAL), tool (TOO), threat actor (THR), campaign (CAM), vulnerability (VUL), mitigation (MIT), attack technique (ATT), group (GRO), consequence (CON), and organization (ORG), as explained in Table 2. The following relations were considered: Target, Attributed_to, Mitigates, HasVulnerability, Uses, Includes, Exploits, Associates, Causes, and Alias_of. In total, thirty-two refined entity–relation categories were obtained by enumerating valid entity-pair types, as shown in Table 3.

### 3.2. Data Enhancement

The scarcity of data, compounded by the extensive array of relationship categories, has given rise to a distinct challenge: the inadequate annotation of data. To address this challenge, the few-shot learning capabilities of large language models were used, with ChatGPT (version GPT-4o) [29] being used to implement a structured data augmentation process [30,31,32].

- **Initial Dataset:** We started with 1051 basic samples.
- **Data augmentation Process:** During data augmentation, an instruction-based rewriting process was constructed around each base sample to increase corpus diversity and robustness. For each sentence, a refined prompt was injected: “Original sentence. Please provide six additional sentences on the same theme that remain similar to the original. The generated sentences must be in English. You may expand content and internal relations using Wikipedia and verified facts, and add new content related to the theme. Entities and relations in the rewritten sentences should not change substantially. All generated sentences must be in lowercase.”
  To balance efficiency and quality, the OpenAI API with the “deepseek” model was initially employed for batch generation to achieve high throughput. However, the outputs did not meet downstream requirements. Considering the cost–quality trade-off, a switch was made to the web-based GPT-4o for manual, interactive rewriting and quality control. Instructions were entered sequentially. Low-level specifications (all-lowercase text and syntactic fluency) were verified. Consistency of entities and relations was validated. Newly introduced facts were rapidly fact-checked to remove semantic drift and hallucinatory samples.
  This procedure markedly improved semantic fidelity and trainability while maintaining controllable generation speed. As a result, enhanced corpora with a higher signal-to-noise ratio were produced for subsequent model training. Representative generated sentences are shown in Table 4.
- **Quality Assurance:** For quality assurance, a semi-automated quality-control scheme was adopted under resource constraints and in the absence of external security reviewers. The scheme was centered on manual verification by researchers with cybersecurity expertise. Each generated sample was comprehensively reviewed and corrected to ensure syntactic fluency, clarity of meaning, and factual accuracy.
  Because the dataset primarily consists of text and entity–relation structures, traditional semantic-similarity measures (e.g., cosine similarity) were deemed inapplicable. An automated screening strategy based on “entity–relation preservation” was therefore developed. A predefined, manually annotated entity–relation dataset was used as the baseline. Normalized definitions of relations were provided to the model, and entity–relation annotations were applied to newly generated sentences. The annotated relations of generated sentences were then compared one-to-one with those of the original samples.
  If core entities and their relations remained unchanged, a sample was deemed semantically consistent and retained. If relation drift or entity mismatch was detected, the sample was flagged as non-compliant and excluded. Structural consistency served as the primary quality metric. Manual review supplemented this metric to correct potential model-annotation errors and factual issues, thereby ensuring semantic fidelity and factual reliability without relying on traditional similarity metrics.
- **Final Dataset:** After enhancement and filtering, we obtained a total of 4741 samples, including the original 1051 samples and 3690 generated samples.

### 3.3. Coding Layer Design for Joint BERT and BiGRU

The primary objective of this layer is to extract context-aware information for each token in a sentence. A Transformer-based bidirectional encoder, BERT, is employed to produce word embeddings [13]. To incorporate threat-intelligence-specific domain knowledge, the embeddings from the BERT layer are further processed by a BiGRU to obtain deep contextual features of the sentence [14].

Given an input sentence $S=\{w_{1},w_{2},...,w_{t},...,w_{l}\}$, where $w_{t}$ is the t-th word in the sentence, in order to better obtain the contextual information of each sequence in the sentence, the pre-trained model BERT is loaded to generate the word embedding vectors, and the outputs are the context-aware word embeddings $E=\{x_{1},x_{2},...,x_{l}\}$, $E\in R^{l\times d}$, where *l* stands for is the length of the sentence (including [CLS] and [SEP]), and *d* is the number of hidden units of the BERT model. The specific formula is shown in (1) below:

(1) E=BERT(S)

To improve model generalization, a BiGRU was employed to extract deep context-aware features for each token. BiGRU extends GRU and operates as a bidirectional Recurrent Neural Network. It is designed to mitigate the long-range dependency limitations of traditional RNNs.

A BiGRU comprises two GRU directions. The forward GRU processes the input from left to right, and the backward GRU processes the input from right to left. Using a BiGRU rather than a unidirectional GRU enables the simultaneous capture of past and future context at each time step, thereby improving sequence feature modeling efficiency. Compared with LSTM, GRU uses two gates—the update gate and the reset gate. This design reduces the number of parameters and improves computational efficiency.

The word embedding vector ***E*** output from the BERT layer is used as an input to the BiGRU network, and the semantic features of the sentence are further extracted to obtain new sequence vectors $H_{B}=\left\{H_{1},H_{2},...,H_{t},...,H_{l}\right\}$, $H_{B}\in R^{l\times d}$, where the output of BiGRU is spliced from the hidden states of the forward and backward GRUs to capture the contextual information of the sequence more comprehensively. The formulas of BiGRU are shown in (2)–(4) below:

(2) $$h_{t}^{f}={\mathrm{GRU}}_{\mathrm{forword}}\left(x_{t},h_{t-1}^{f}\right)$$

(3) $$h_{t}^{b}={\mathrm{GRU}}_{\mathrm{backword}}\left(x_{t},h_{t+1}^{b}\right)$$

(4) $$H_{t}=\left[h_{t}^{f};h_{t}^{b}\right]$$

where $x_{t}$ is the feature representation of the t-th input token, $h_{t-1}^{f}$ is the hidden state of the (t − 1)-th input token, $h_{t+1}^{b}$ is the hidden state of the (t+1)-th input token, $h_{t}^{f}$ represents the hidden state of the forward GRU unit, $h_{t}^{b}$ represents the hidden state of the backward GRU, and $H_{t}$ represents the final result of the current sequence after one layer of BiGRU network.

### 3.4. Parallel Decoding Layer Design

Following [22], a non-autoregressive decoder (NAD) is employed, which formulates joint entity–relation extraction as an ensemble prediction problem. Unlike autoregressive decoders that condition on previously generated outputs, the NAD uses maskless self-attention to access information at all sequence positions simultaneously. Triples are therefore generated in parallel, which markedly improves efficiency. For each sentence, the decoder outputs a set of N fixed-size predicted triples. The value of N is set greater than the maximum number of triples present in the sentence. The query vector $I_{Q}$ is initialized with N shared, learnable embeddings and is computed by Equation (5).

(5) $$I_{Q}=\mathrm{IniEmbedding}\left(N\right)$$

The core of the non-autoregressive decoder consists of M Transformer blocks. Each block comprises three sublayers: multi-head self-attention, multi-head cross-attention, and a feed-forward network. The multi-head self-attention sublayer models intra-triple dependencies. Its output projection is used as the query (***Q***) for the multi-head cross-attention sublayer. The encoder outputs provide the keys (***K***) and values (***V***) for that cross-attention, thereby fusing sentence-level information. The computations of the multi-head cross-attention sublayer are given by Equations (6)–(8).

(6) $$\begin{array}{cc} {\hat{H}}_{D} & =MultiHead(H_{D},H_{B},H_{B})\\ & =Concat\left(head_{1},...,head_{h}\right)W^{0} \end{array}$$

(7) $$head_{i}=Attention\left(H_{D}W_{i}^{Q},H_{B}W_{i}^{K},H_{B}W_{i}^{V}\right)$$

(8) $$Attention\left(Q,K,V\right)=\mathrm{softmax}\left(\frac{{\mathrm{QK}}^{T}}{\sqrt{d_{k}}}\right)V$$

$H_{D}$ is the output of the previous multi-head self-attention sublayer, and $H_{B}$ is the output of the encoder. $W^{0}$, $W_{i}^{Q}$, $W_{i}^{K}$, $W_{i}^{V}$ are trainable parameters, *h* is the number of attention heads, $d_{k}$ is the dimensional size of the key vector. The score can be scaled by dividing by $\sqrt{d_{k}}$, which helps to enhance the stability of the gradient. The multi-head self-attention sublayer has the same formulas (6)–(8), unlike the multi-head cross self-attention sublayer, whose ***Q***, ***K***, and ***V*** are initialized learnable embeddings. With the non-autoregressive decoder, the N ternary queries are converted into N output embeddings, which are denoted as $E_{T}\in R^{N\times d}$.

Finally, the output embedding $E_{T}$ is inputted into the MLP network to decode the entities and relations independently, respectively, and the MLP network predicts to get the relation label $P^{r}$, as well as the start position and end position of the head and tail entities $P^{h-start}$, $P^{h-end}$, $P^{t-start}$, and $P^{t-end}$ by the softmax classifier. The specific equations are shown in (9), (10)–(13) below:

(9) $$P^{r}=\mathrm{softmax}\left(W_{r}E_{T}\right)$$

(10) $$P^{h-start}=\mathrm{softmax}\left(V_{1}^{T}\tanh\left(W_{1}E_{T}+W_{2}H_{B}\right)\right)$$

(11) $$P^{h-end}=\mathrm{softmax}\left(V_{2}^{T}\tanh\left(W_{3}E_{T}+W_{4}H_{B}\right)\right)$$

(12) $$\begin{array}{c} P^{t-start}=\mathrm{softmax}\left(V_{3}^{T}\tanh\left(W_{5}E_{T}+W_{6}H_{B}\right)\right) \end{array}$$

(13) $$\begin{array}{c} P^{t-end}=\mathrm{softmax}\left(V_{4}^{T}\tanh\left(W_{7}E_{T}+W_{8}H_{B}\right)\right) \end{array}$$

where ${\left\{V_{i}^{T}\in R^{d}\right\}}_{i=1}^{4}$ and ${\left\{W_{i}\in R^{d\times d}\right\}}_{i=1}^{8}$ are the learnable parameters and $H_{B}$ is the output of BiGRU encoder based on BERT to obtain the final prediction triples as $\hat{T}=\left(\begin{array}{c} P^{r},P^{h-start},P^{h-end},P^{t-start},P^{t-end} \end{array}\right)$.

### 3.5. Loss Function

For model optimization, the Bipartite Matching Loss (BMLoss) was employed [22]. Optimal alignments between predicted and gold triples were obtained with the Hungarian algorithm. The loss comprises relation classification and entity position prediction, both computed via negative log-likelihood.

## 4. Experiments

### 4.1. Datasets

**Figure 2:**
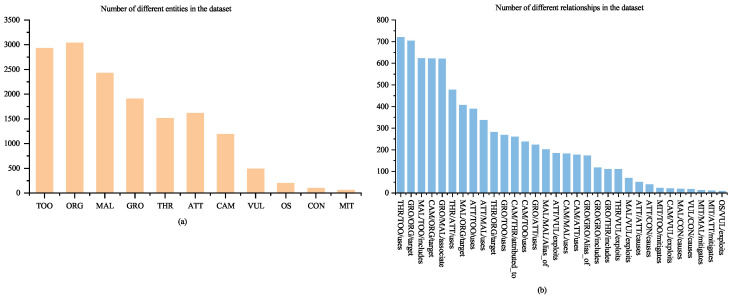
Distribution of the number of entities and relationships: (**a**) Number of different entities in dataset; (**b**) Number of different relationship in the dataset.

This study collects a total of 1489 publicly available security reports related to APT organizations and cybercriminal activities [33], which cover organizations or operations active from 2008 to July 2024, from which 36 high-quality reports are filtered based on the keyword density of cybersecurity terminology [12]. These reports are mainly related to APT organizations and malware families, such as APT37, APT10, and Andromeda. After the data cleaning and preprocessing work, a brand new dataset SecCti is constructed, and a total of 4741 sentences are obtained. A total of 15,474 entities and 7699 relationships are labeled, and the distribution of the number of entities and relationships is shown in Figure 2. The data is divided into training set, evaluation set, and test set according to an 8:1:1 ratio. The pattern of overlapping relationship triples in the dataset is shown in Table 5.

### 4.2. Experimental Setup

- **Implementation Details:** In this study, PyTorch (version 2.7.0) was used to create the experimental models and all experiments were conducted using NVIDIA GeForce RTX 4090 Ti. In the BERT-based BiGRU sentence encoder used in this paper, the BERT word embedding contains 100M parameters, the initial dimension of the word embedding representation is 768, the dimension of the BiGRU is 256, and 3 layers of BiGRU are set up with an initial learning rate of 0.00002. The number of non-autoregressive decoder layers is set up as 3 layers with an initial learning rate of 0.00005, and a total of 100 rounds of training are performed. The coding layer discard rate is set to 0.5 to prevent the model from overfitting during training.
- **Evaluation Metrics:** In this paper, standard micro-average precision (Precision), micro-average recall (Recall), and micro-average F1 (F1) score are used to evaluate the performance of the model. A triple is considered to be correctly extracted when and only when its relation type and two entities are correctly matched.

(14) $$P_{micro}=\frac{\sum_{c=1}^{C}TP_{c}}{\sum_{c=1}^{C}\left(TP_{c}+FP_{c}\right)}$$

(15) $$recall_{micro}=\frac{\sum_{c=1}^{C}TP_{c}}{\sum_{c=1}^{C}\left(TP_{c}+FN_{c}\right)}$$

(16) $$F1_{micro}=2*\frac{P_{micro}*recall_{micro}}{P_{micro}+recall_{micro}}$$

where *C* is the total number of relation categories, $TP_{c}$ is the number of True Positives for category *c*, $FP_{c}$ is the number of False Positives for category *c*, and $FN_{c}$ is the number of False Negatives for category *c*. A triple is considered to be a true positive instance when both entities and relations extracted are correct.

### 4.3. Results and Analysis

#### 4.3.1. Baseline Model

CyberRel [11]: A multiple-sequence labeling model for the joint extraction of cybersecurity conceptual entity–relationships is used. Separate sequences of tags are generated for different relationships containing information about the entities involved, as well as the subject and object of the relationship. BERT-BiLSTM-CRF [20]: the end-to-end sequential annotation model of BERT-att-BiLSTM-CRF is used to realize the joint extraction of entities and relations, and finally the knowledge triples are extracted according to the rules for matching entities and relations. CyberEntRel [12]: utilizes the attention-based RoBERTa-BiGRU-CRF model for sequential labeling. After matching the most appropriate relationship for two predicted entities, the relationship triples are extracted using the relationship matching technique.

#### 4.3.2. Comparison Experiments

In order to validate the effectiveness of the model, the performance of this paper is compared with three baseline models. As shown in Table 6, the model proposed in this paper outperforms the other methods on both the overall relationship triple extraction (All) and the overlapping entity relationship triple extraction (Overlap) tasks. Compared with the state-of-the-art CyberEntRel method, the F1 scores of the two tasks are improved by 4.6% and 9.6%, respectively.

The performance improvement is mainly due to two aspects: first, the BiGRU encoder is able to capture deep contextual information effectively; second, the non-autoregressive decoder adopts a mask-free self-attention mechanism, which can process all the positional information in parallel and generate the set of relational triples directly. In addition, a high recall rate is ensured by setting an adequate number of ternary queries, which enables the model to achieve a more balanced performance in terms of both precision and recall.

It is worth noting that, in the domain-specific task of this paper, better results were achieved using the BiGRU network with fewer parameters and simpler structure than BiLSTM. This may be due to the fact that on smaller and noisy datasets, the simplified structure of BiGRU helps to reduce the risk of overfitting while improving the computational efficiency.

#### 4.3.3. Ablation Experiments

The importance of the BiGRU and the effect of decoder depth were assessed via ablation experiments. Results are reported in Table 7. BiGRU depths of 1, 2, 3, 4, 5, and 6 layers were evaluated. For depths below three, model capacity and expressive power increased with depth. With three BiGRU layers, the model achieved the best performance: the F1 score improved by 5.6% relative to a model without BiGRU, demonstrating the effectiveness of the BiGRU subnetwork. Beyond three layers, performance declined. The degradation is attributed to overfitting to training noise and unnecessary complexity introduced by deeper architectures. Decoder depth was analyzed in the same manner. When the number of decoder layers was below three, performance improved with additional layers. The highest F1 score was obtained at three layers, after which performance decreased. Although the non-autoregressive decoder supports parallel decoding, excessive depth may prevent the full utilization of contextual information, leading to diminishing returns.

#### 4.3.4. Hyperparametric Experiments

**Figure 3:**
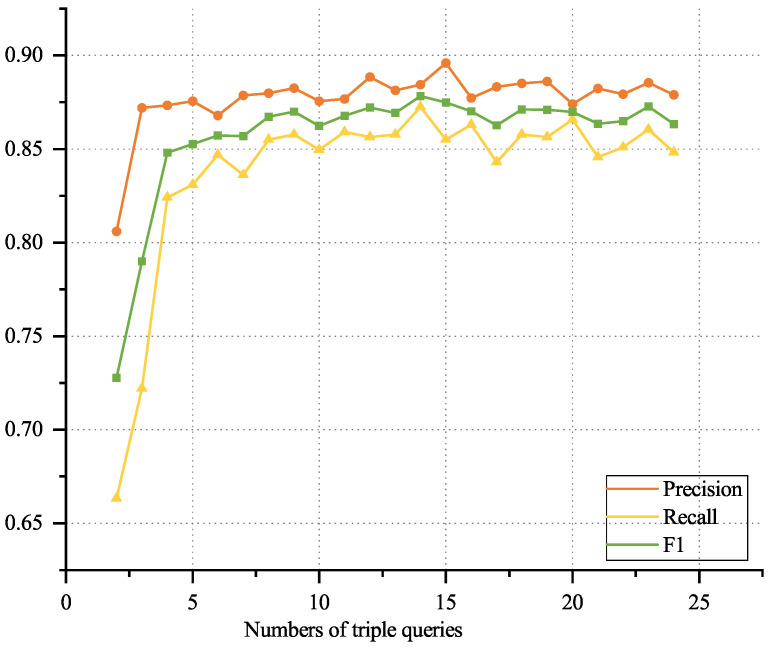
Variation of model performance with different numbers of triple queries.

For this dataset, the effect of varying numbers of triple queries (2–24) on model performance was evaluated, as shown in Figure 3. Performance improved as the number of queries increased up to 14, at which point the highest F1 score was achieved. Beyond 14 queries, performance gradually decreased, likely due to interference from excessive invalid triple candidates.

#### 4.3.5. Visual Analysis

**Figure 4:**
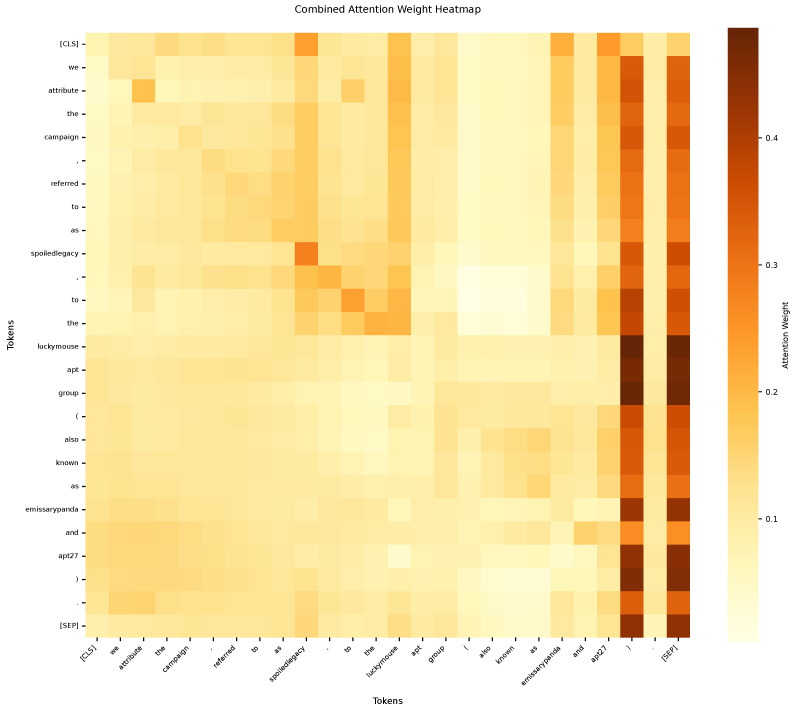
Heat map of encoder attention weights.

To provide an intuitive view of the model’s mechanism, the attention distributions of the encoder and decoder were visualized. As shown in Figure 4, the encoder—comprising BERT and BiGRU—accurately identifies key entity tokens such as “spoiledlegacy,” “APT27,” and “EmissaryPanda.” Clear attention focus regions are also formed around threat-intelligence entities such as “LuckyMouse,” “APT,” and “group.” These patterns indicate that task-relevant semantic features have been effectively captured by the model.

**Figure 5:**
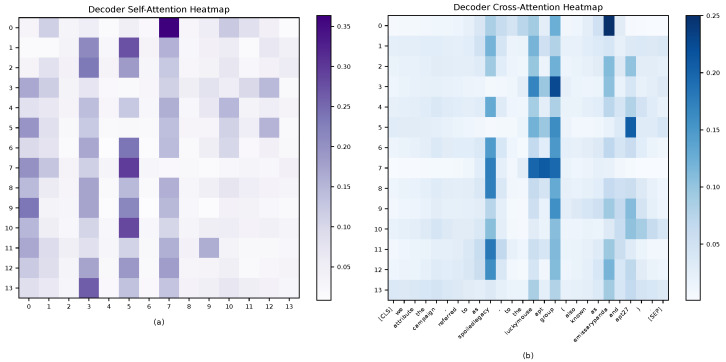
Heat map of decoder self-attention and cross-attention weights: (**a**) Self-attention weights between decoder queries; (**b**) Cross-attention weights from decoder queries to input entities.

Figure 5 presents the decoder’s self-attention and cross-attention distributions. In the self-attention heat map (Figure 5a), query positions 3, 5, and 7 form a clear high-attention region. Diagonal weights are generally low, indicating information sharing across query vectors. In the cross-attention heat map (Figure 5b), distinct entity focuses are observed. Query 3 attends to “LuckyMouse APT group” and “EmissaryPanda.” Query 5 attends to “LuckyMouse APT group” and “APT27.” Query 7 attends to “LuckyMouse APT group” and “SpoiledLegacy.” These patterns suggest that overlapping relations are handled efficiently. Different query vectors focus on different entity pairs in parallel while sharing key entity information.

#### 4.3.6. Case Study

**Figure 6:**
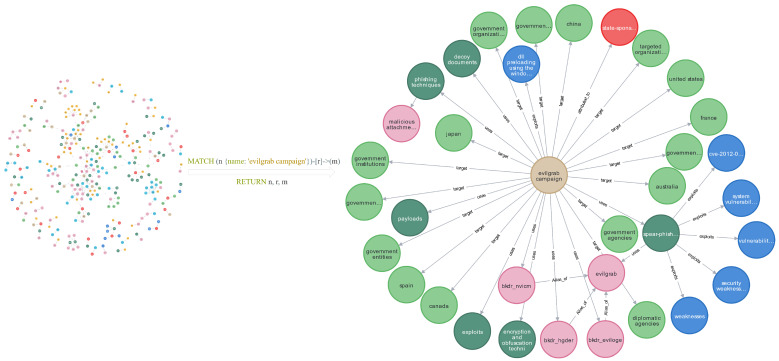
Example of cyber threat intelligence knowledge graph.

The cyber threat intelligence (CTI) extracted in this study was imported into a Neo4j graph database. The database contains 2552 entities and 2680 relations. A partial view of the CTI knowledge graph is shown in Figure 6. Information related to the EvilGrab campaign was queried using Cypher to obtain detailed context. The results indicate that the EvilGrab campaign primarily targets countries such as China and Canada, along with their governmental organizations. The campaign employs harpoon-style network attacks using the EvilGrab malware and primarily exploits vulnerabilities such as CVE-2012-0158. These details provide multi-dimensional insight into the attack. Based on this information, security analysts can specify more effective defensive strategies against cyberattacks.

## 5. Conclusions and Future Work

In this study, a joint extraction method for CTI entity–relation triples based on parallel ensemble prediction was proposed. A BERT–BiGRU encoder was combined with a non-autoregressive decoder to capture deep context and to generate triples in parallel. Competitive performance was achieved on SecCti, with clear gains on overlapping triples. Building on these results, key advantages, limitations, comparisons, and future directions are outlined below:

1. Advantages: First, order-dependency and exposure-bias issues are mitigated by set-style, non-autoregressive decoding. Parallel decoding improves efficiency on inputs with multiple triples. A BiGRU layer provides lightweight sequential inductive bias atop BERT, benefiting noisy CTI text.
2. Limitations: Nevertheless, several challenges were identified. Performance is sensitive to the number of query slots; excessive slots yield invalid candidates and may reduce F1. Deeper encoders/decoders risk overfitting, requiring careful depth and regularization. Accurate span localization remains critical, and domain drift and label noise can affect robustness. Moreover, the present study comprised a total of 36 reports, 4741 sentences, and 7699 relationships. It is acknowledged that the current body of evidence is limited in terms of both sources and scale, a factor which may have a bearing on the universality of the findings across institutions and time periods.
3. Comparison: In relation to prior approaches, error propagation is reduced via joint decoding compared with pipeline NER→RE. Relative to joint sequence-labeling baselines (e.g., BERT-BiLSTM-CRF-style), overlapping triples are handled more effectively and decoding is faster, though sequence-labeling can be simpler for single-triple cases.
4. Future work: Emphasis will be placed on document-level Chinese CTI, adaptive query allocation, boundary-aware targeting, noise-aware training, and enhanced Neo4j governance. In addition, as resources increase, the scope of evaluation will be expanded by introducing CTI reports from more institutions and time periods to improve source diversity, and the automation of end-to-end CTI knowledge graph construction based on large models will be explored.

## Figures and Tables

**Table 1:** Example of threat intelligence information.

	Texts	Triples
**Normal Example**	apt20 regularly employs mimikatz to obtain credentials from accounts with elevated privileges	(apt20, THR/TOO/uses, mimikatz)
**Overlapping Example**	we attribute the campaign, referred to as spoiledlegacy, to the luckymouse apt group (also known as emissarypanda and apt27)	(spoiledlegacy, CAM/THR/attributed_to, **luckymouse apt group**), (emissarypanda, GRO/GRO/Alias_of, **luckymouse apt group**), (apt27, GRO/GRO/Alias_of, **luckymouse apt group**)

**Table 2:** Entity Categories.

Type of Entity	A Concrete Explanation
MAL	Harmful programs or viruses
TOO	Security test and attack equipment
TH	Threat actors who launch cyberattacks
CAM	Cyberattack or incident
ATT	Methods and strategies for launching attacks
GRO	Organization of the team that carried out the attack
CON	Impact or damage from cyber incidents
ORG	Organizations likely to be attacked
OS	Basic software environment for equipment
VUL	Security deficiencies in the system
MIT	Risk-reducing security programs

**Table 3:** Relationship categories.

Part 1			Part 2		
**Head**	**Relationship**	**Tail**	**Head**	**Relationship**	**Tail**
CAM	CAM/ORG/target	ORG	MAL	MAL/MAL/Alias_of	MAL
THR	THR/TOO/uses	TOO	ATT	ATT/VUL/exploits	VUL
GRO	GRO/ORG/target	ORG	CAM	CAM/MAL/uses	MAL
MAL	MAL/TOO/includes	TOO	CAM	CAM/ATT/uses	ATT
GRO	GRO/MAL/associate	MAL	GRO	GRO/GRO/Alias_of	GRO
THR	THR/ATT/uses	ATT	GRO	GRO/GRO/includes	GRO
MAL	MAL/ORG/target	ORG	GRO	GRO/THR/includes	THR
ATT	ATT/TOO/uses	TOO	HTR	THR/VUL/exploits	VUL
ATT	ATT/MAL/uses	MAL	MAL	MAL/VUL/exploits	VUL
THR	THR/ORG/target	ORG	ATT	ATT/ATT/causes	ATT
GRO	GRO/TOO/uses	TOO	ATT	ATT/CON/causes	CON
CAM	CAM/THR/attributed_to	THR	MIT	MIT/TOO/mitigates	TOO
CAM	CAM/TOO/uses	TOO	CAM	CAM/VUL/exploits	VUL
GRO	GRO/ATT/uses	ATT	MAL	MAL/CON/causes	CON
VUL	VUL/CON/causes	CON	MIT	MIT/MAL/mitigates	MAL
OS	OS/VUL/exploits	VUL	MIT	MIT/ATT/mitigates	ATT

**Table 4:** Comparison of raw and GPT-generated data.

Raw Data	GPT Post-Generation Data
During the same time period, we also observed the actor using the browser exploitation framework (beef) to compromise victim hosts and download cobalt (aka “beef”) strike.	Concurrently, we observed the actor making use of the browser exploitation framework (beef) to infiltrate victim machines and pull down cobalt strike.
During this infection process, the actor was known to check the target operating system and deliver malware, signed by a previously stolen key, for the appropriate host environment.	During this infection period, the actor was known to inspect the target OS and deliver malware, which was signed using a key that had been stolen earlier.

**Table 5:** Triples overlap mode.

Dataset	Normal	Overlap	All
training set	2548	3598	6146
validation set	291	454	745
test set	306	502	808

**Table 6:** Experimental results of different models.

Model	All Triples			Overlap Triples		
	Precision/%	Recall/%	F1/%	Precision/%	Recall/%	F1/%
CyberRel [11]	83.0	79.1	80.9	-	-	-
BERT-BiLSTM-CRF [20]	82.1	70.4	75.8	77.9	67.2	72.1
CyberEntRel [12]	89.4	77.8	83.2	85.2	74.6	79.5
our model (BiLSTM-NAR)	87.7	86.5	87.1	90.6	85.9	88.2
our model (BiGRU-NAR)	88.4	87.2	87.8	91.4	87.0	89.1

**Table 7:** Ablation experiment results.

Model	Precision/%	Recall/%	F1/%
our model	88.4	87.2	87.8
No-BiGRU	86.2 (−2.2)	84.2 (−3.0)	82.2 (−5.6)
6-BiGRU	86.8 (−1.6)	85.4 (−1.8)	86.1 (−1.7)
5-BiGRU	88.1 (−0.3)	86.4 (−0.8)	87.3 (−0.5)
4-BiGRU	88.2 (−0.2)	85.2 (−2.0)	86.7 (−1.1)
2-BiGRU	87.5 (−0.9)	85.4 (−1.8)	86.4 (−1.4)
1-BiGRU	88.5 (+0.1)	85.6 (−1.6)	87.0 (−0.8)
6-NAD	88.1 (−0.3)	86.2 (−1.0)	87.2 (−0.6)
5-NAD	88.5 (+0.1)	85.7 (−1.5)	87.1 (−0.7)
4-NAD	87.5 (−0.9)	85.6 (−1.6)	86.6 (−1.2)
2-NAD	87.6 (−0.8)	86.3 (−0.9)	86.9 (−0.9)
1-NAD	86.8 (−1.6)	85.5 (−1.7)	86.1 (−1.7)

## Data Availability

The original contributions presented in the study are included in the article; further inquiries can be directed to the corresponding author.

## Abbreviations

The following abbreviations are used in this manuscript:

DDoS	Distributed Denial-of-Service
BERT	Bidirectional Encoder Representations from Transformers
RNN	Recurrent Neural Network
CRF	Conditional Random Field
BiGRU	Bidirectional Gated Recurrent Unit
BiLSTM	Bidirectional Long Short-Term Memory
ChatGPT	Chat Generative Pre-trained Transformer
NAD	Non-autoregressive Decoder
APT	Advanced Persistent Threats
NER	Named Entity Recognition
RE	Relation Extraction
NLP	Natural Language Processing
MLP	Multilayer Perceptron
